# Estimates of genetic parameters for chemical traits of meat quality in Japanese black cattle

**DOI:** 10.1111/asj.12622

**Published:** 2016-05-05

**Authors:** Hironori Sakuma, Kaoru Saito, Kimiko Kohira, Fumie Ohhashi, Noriaki Shoji, Yoshinobu Uemoto

**Affiliations:** ^1^ National Livestock Breeding CenterNishigoFukushimaJapan; ^2^ Yamagata Prefectural College of AgricultureShinjoYamagataJapan

**Keywords:** *carcass trait*, *chemical trait*, *genetic parameters*, *Japanese Black cattle*, *water‐soluble compounds*

## Abstract

Genetic parameters for 54 carcass and chemical traits, such as general composition (moisture, crude fat and crude protein), fatty acid composition and water‐soluble compounds (free amino acids, peptides, nucleotides and sugars) of 587 commercial Japanese Black cattle were assessed. Heritability estimates for carcass traits and general composition ranged between 0.19–0.28, whereas those for fatty acid composition ranged between 0.11–0.85. Most heritability estimates for water‐soluble compounds were lower than 0.30; these traits were affected by aging period. Moderate heritability was observed for glutamine, alanine, taurine, anserine, inosine 5′‐monophosphate (IMP), inosine and *myo*‐inositol. In particular, heritability estimates were the highest (0.66) for taurine. Traits with moderate heritability were unaffected by aging period, with the exception of IMP, which was affected by aging period but exhibited moderate heritability (0.47). Although phenotypic correlations of water‐soluble compounds with carcass weight (CW), beef marbling standard (BMS) and monounsaturated fatty acid were generally low, genetic correlations between these traits were low to high. At the genetic level, most of the water‐soluble compounds were positively correlated with monounsaturated fatty acid but negatively correlated with CW and BMS. Thus, our results indicate that genetic variance and correlations could exist and be captured for some of the water‐soluble compounds.

## Introduction

Intramuscular crude fat (CF) content directly influences the quality of meat and is therefore one of the most economically important traits in beef cattle breeding (Iida *et al.* 2015). Although CF content is related to the tenderness and juiciness of meat, studies have shown that consumers do not necessarily favor excessive marbling (Iida *et al.* 2015). The ‘eating quality’ of meat is largely determined by the sensory characteristics such as taste, tenderness, juiciness and aroma. In addition to CF content, other factors have been reported as affecting the eating quality of meat, including fatty acid composition (David 2008; Sakuma *et al.* 2012). In addition, beef palatability has been shown to be related to water‐soluble compounds, such as free amino acids (e.g. glutamic acid, Glu, and aspartic acid, Asp; Kawai *et al.* 2002), peptides (e.g. carnosine, Car; Mateescu *et al.* 2012) and nucleotides (e.g. inosine 5′‐monophosphate, IMP; Kawai *et al.* 2002), as well as sugars (e.g. glucose, Glc, and fructose, Fru). Sugars could contribute to the sweetness (MacLeod 1994) and flavor of meat upon cooking, due to the Maillard reaction (Mottram 1998). In recent years, consumer demand for healthier meat has grown but without compromising on the eating quality of meat. The physiological activity of several meat‐based bioactive substances, such as conjugated linoleic acid, Car, anserine (Ans) and taurine (Tau), have been thoroughly examined (Arihara 2004). Improvements in the integration of these bioactive compounds in meat constitute one possible approach to meet rising consumer demand for healthier meat. Thus, to improve the taste and nutritional value of meat, it is necessary to first evaluate chemical traits such as moisture (MOIS), CF, crude protein (CP), fatty acid composition, free amino acids, peptides, nucleotides and sugars.

Improvements in the chemical traits of meat can be achieved through manipulation of both genetic and environmental factors (e.g. diet, aging and feed system). Genetic effects in some traits have been reported; for example, fatty acid composition is a heritable trait, with heritability ranging between 0.31 and 0.73 in the Trapezius muscle (Inoue *et al.* 2008) and between 0.58 and 0.78 in the Longissimus muscle (Nogi *et al.* 2011) of Japanese Black cattle. However, most studies have focused on the fatty acid composition of meat; to our knowledge, only one study (Mateescu *et al.* 2012) has investigated the low to moderate heritabilities of peptides in Angus cattle. Thus, information regarding the genetic effects of other chemical traits in beef cattle is generally lacking. Therefore, more comprehensive knowledge on the chemical traits of meat is necessary prior to improvement in meat quality through genetic manipulations.

The objective of the present study was to evaluate the influence of genetic factors on meat quality traits by estimating the genetic parameters for a number of carcass and chemical traits, including MOIS, CF, CP and fatty acid composition, as well as amino acid, peptide, nucleotide, and sugar contents in Japanese Black cattle.

## Materials and methods

### Animals, carcass traits and sample collection

Approval from the Animal Care and Use Committee was not obtained for the present study because the data were collected from beef cattle shipped to a meat processing plant in Yamagata Prefecture, Japan.

A population of commercial Japanese Black cattle reared in Yamagata Prefecture, Japan, was used in our study (Sasago *et al.* 2016). The rib loin was resected from 587 Japanese Black cattle slaughtered between 2011 and 2013 at a meat processing plant in the Yamagata Prefecture. Each carcass was graded at the level of the sixth to seventh rib by official Japanese graders according to the Japanese Grading Standards (JMGA 1989), which is based on the yield (A: above average, B: average and C: below average) and meat quality grades (1: poor to 5: excellent). Of the 587 carcasses we obtained, the grades A3, A4, A5, B3, B4and B5 were assigned to 230, 246, 91, nine, 10 and one carcasses, respectively. Carcass traits that were analyzed consisted of carcass weight (CW, in kg) and beef marbling standard (BMS, ranging from 1 (poor) to 12 (very abundant)).

Approximately 1 kg of rib loin at the seventh thoracic vertebra was frozen for 16–19 days after slaughter and was purchased from a distributor. The rib loin was thawed at 2°C for 16 h, and the Longissimus thoracis muscle was sliced into strips of 1 cm thickness to be used for the assessment of MOIS, CF and CP, and into strips of 2 mm thickness for the assessment of fatty acid composition, as well as free amino acid, peptide, nucleotide and sugar contents. Samples for the assessment of MOIS, CF and CP were analyzed on the day of thawing, whereas samples for the assessment of fatty acid composition and free amino acid, peptide, nucleotide and sugar contents were stored at −30°C until the time of analysis. All of the slices were minced before performing the analyses; all analyses were carried out in duplicate.

### Measurement of general composition (MOIS, CF and CP contents) and fatty acid composition

For the purposes of the present study, MOIS, CF and CP contents were referred to as the ‘general composition’ and were measured according to the procedures described by Okumura *et al.* (2012). MOIS content was determined in duplicate by drying 2 g samples of meat drawn from the minced raw meat (about 30 g in total) for 24 h at 105°C. CF content was determined by Soxhlet extraction of the dried samples, obtained by analyzing the MOIS content with diethyl ether for 16 h. CP content was determined using 1 g samples of meat drawn from the minced raw meat (about 30 g in total) using the Kjeldahl method using a nitrogen distillation titration device (2400 Kjeltec Auto Sampler System; FOSS, Hillerød, Denmark).

Total lipid extraction was performed using approximately 0.5 g samples of meat drawn from the minced raw meat (about 15 g in total), in accordance with the procedures described by Folch *et al.* (1957) for analyzing fatty acid composition. The lipids were methylated using 0.5 N sodium methoxide in methanol and extracted into hexane. The lipid was then analyzed using a gas chromatograph (GC, model 6890A; Agilent Technologies Inc., Santa Clara, CA, USA) equipped with a flame ionization detector and a capillary column (TC‐70, 60 m in length, internal diameter (i.d.) of 0.25 mm, film thickness of 0.25 µm; GL Science, Tokyo, Japan) with the injector set at 240°C in split mode (10:1) and the detector at 250°C, and the oven temperature program held at 190°C for 8 min following injection, then increased to 230°C at a rate of 20°C/min and held at 230°C for 2 min, with a total acquisition program of 12 min. Helium was used as the carrier gas, with a flow rate of 1.8 mL/min. Fatty acids were identified through comparison of their retention times with those of established standards, and fatty acids were expressed as percentages of the total fatty acid content.

### Measurement of free amino acids, peptides and nucleotides

Extractions of free amino acids, peptides and nucleotides were performed using approximately 0.10 g samples of meat drawn from the minced raw meat (about 15 g in total). Samples were homogenized with 4.24 mL of ultrapure water, 4 mL of N‐hexane and 0.16 mL of internal standard solution mixed with norvaline (5 nmol/μL) and cytidine (5 nmol/μL) in ultrapure water, and then centrifuged at 1750 × *g* for 5 min. The underlayer was mixed with 4 mL of N‐hexane and centrifuged at 1750 × *g* for 5 min. The resultant underlayer was then mixed with 3.6 mL of acetonitrile and centrifuged at 1750 × *g* for 10 min. The resulting supernatant was filtered through a 0.45‐µm microfilter (Millex‐LH; Merck Millipore, Billerica, MA, USA), and the filtrate was then mixed with 45% acetonitrile solution and analyzed for free amino acids and peptides using an Agilent 1260 infinity high performance liquid chromatograph (HPLC) equipped with an Agilent 1260 Binary Pump, 1260 HiP Degasser, 1260 HiP ALS autosampler, 1290 thermostat, 1260 Thermostatted column compartment (TCC) control module, 1260 diode array detector (DAD) and a Poroshell 120 EC‐C18 column (3.0 × 100 mm, 2.7 µm; Agilent). The eluents used were: (i) 20 mMmol/L disodium hydrogen phosphate (pH 7.6); and (ii) acetonitrile/methanol/water (5:5:1, v/v/v). To analyze nucleotide contents, 20 μL of the filtrate was mixed with 180 μL of ultrapure water, with the resulting solution analyzed using the HPLC (Waters 2695; Waters, Milford, MA, USA) along with an Atlantis T3 column (4.6 × 150 mm, 5 µm; Waters) and a UV detector (Waters 2487; Waters). The eluent used was 100 mmol/L potassium dihydrogen phosphate (pH 4.0). As with fatty acids, amino acids, peptides and nucleotides were identified through comparison of their retention times with those of established standards. The concentrations of each were calculated using internal and external standard solutions, and expressed as µmol per g of meat and µmol per 1% of moisture in meat and moisture, respectively. The internal standard solutions were used to account for matter lost during analysis, and the external standard solutions (1, 10, 50 and 100 pmol/μL) were used to plot a calibration curve for each amino acid, peptide and nucleotide.

### Measurement of sugars

Sugar extraction was performed using approximately 0.25 g samples of meat drawn from the minced raw meat (about 15 g in total). Each sample was homogenized with 5 mL of N‐hexane and 1.5 mL of xylose solution (1 mg/mL) as the internal standard, and centrifuged at 1750 × *g* for 5 min. The underlayer was mixed with 5 mL of N‐hexane and centrifuged at 1750 × *g* for 5 min, then mixed with 3.5 mL of acetonitrile and centrifuged at 1750 × *g* for a further 10 min. For analysis by GC, 1.5 mL of the supernatant and 70% acetonitrile solution were mixed with 2 mL of ethanol, following which the solution was allowed to evaporate. The residue was re‐extracted using 300 μL of N‐trimethylsilylimidazole‐H (TMSI‐H) (GL Sciences, Tokyo, Japan) and silylated for 20 min in a water bath at 80°C. The silylated solution was then centrifuged at 1750 × *g* for 5 min. The supernatant was analyzed by a gas chromatograph (GC‐2014; Shimadzu Co. Ltd., Tokyo, Japan) equipped with a flame ionization detector and a capillary column InertCap‐1701 (30 m in length, 0.25 mm i.d., 0.25 µm film thickness; GL Sciences) with the injector set at 280°C in split mode (10:1) and the detector set at 280°C; the oven temperature program was initially set to 151°C and subsequently increased to 181°C at a rate of 5°C/min, and held at 181°C for 17 min; the temperature was further increased to 231°C at a rate of 10°C/min, and held at 231°C for 3 min, for a total acquisition program of 31 min. Helium was used as the carrier gas, with a flow rate of 1.59 mL/min. Sugar contents were identified by comparing retention times with those of established standards. Concentrations of sugar were calculated using internal and external standards, and expressed as µmol per g of meat and µmol per 1% of moisture in meat and moisture, respectively. The internal standards were used to account for what was lost during analysis, and the external standards were used to plot calibration curves for each sugar.

### Statistical analysis

Fifty‐four carcass and chemical traits were analyzed, which included carcass characteristics (two traits), general composition (three traits), fatty acid composition (13 traits), free amino acids (22 traits), peptides (three traits), nucleotides (four traits) and sugars (seven traits). The details of these traits are presented in Tables 1 and 2. Phenotypes that were not within the mean ± 3 standard deviations (SD) for each trait were considered outliers and omitted. The heritabilities of all traits were estimated by the following single‐trait animal model:

$$y_{\text{ikjlmn}}=\mu +sex_{i}+year_{j}+\text{mont}h_{k}+\text{agin}g_{l}+\text{far}m_{m}+b_{1}x_{\text{ikjlmn}}+b_{2}x_{\text{ikjlmn}}^{2}+u_{\text{ikjlmn}}+e_{\text{ikjlmn}}$$

where *y_ijklmn_* is the observation of the animal *n* for the assessed traits; *μ* is the total mean; *sex_i_* is the fixed effect of sex *i* (two classes); *year_j_* is the fixed effect of the slaughter year *j* (three classes, 2011–2013); *month_k_* is the fixed effect of the slaughter month *k* (12 classes); *aging_l_* is the fixed effect of aging period *l* (four classes, 16–19 days); *farm_m_* is the fixed effect of the farm *m* (13 classes); $b_{1}x_{\text{ikjlmn}}+b_{2}x_{\text{ikjlmn}}^{2}$ is linear (*b_1_*) and quadratic (*b_2_*) regression coefficients on slaughter age (*x_ijklm_*); *u_ijklmn_* is the genetic effect of the animal *n*, which is distributed as $N\left(0,A\sigma_{u}^{2}\right)$ (where **A** represents the numerator relationship matrix and $\sigma_{u}^{2}$ is additive genetic variance); and *e_ijklmn_* is the residual effect. Pedigrees of the carcass were traced back five generations to create the numerator relationship matrix, with 3513 animals included in the pedigree analysis. For water‐soluble compounds, the fixed effect of aging period was also tested for significance by an incremental Wald F statistics analysis using the above model. In addition, pairwise bivariate analyses were performed to estimate (co)variance components, and the phenotypic and genetic correlations were calculated. The multi‐trait animal model was applied with the aforementioned fixed and random effects. ASReml 3.0 software (Gilmour *et al.* 2009) was used to test for significance of aging period and to estimate (co)variance components with standard errors.

**Table 1:** Descriptive statistics and heritability of carcass traits, general compositions and fatty acid compositions

Traits	Abbreviations	Unit	*n*	Mean	SD	Min	Max	Genetic variance	Heritability
Carcass traits									
Carcass weight	CW	kg	585	419	48	286	537	530	0.28 ± 0.12
Beef marbling standard	BMS	1 to 12	581	5.89	1.56	3.00	10.00	0.50	0.22 ± 0.12
General compositions									
Moisture	MOIS	%	585	45.86	4.62	32.50	58.00	4.90	0.24 ± 0.13
Crude fat	CF	%	585	39.83	6.23	24.50	57.60	9.40	0.25 ± 0.13
Crude protein	CP	%	586	13.66	1.57	9.10	17.90	0.46	0.19 ± 0.12
Fatty acid compositions									
Myristic acid	C14:0	%	582	2.60	0.46	1.20	4.00	0.12	0.56 ± 0.17
Myristoleic acid	C14:1	%	583	0.99	0.26	0.40	1.80	0.06	0.85 ± 0.18
Palmitic acid	C16:0	%	584	25.84	1.88	21.30	31.10	1.78	0.48 ± 0.17
Palmitioleic acid	C16:1	%	585	4.43	0.68	2.90	6.40	0.38	0.82 ± 0.18
Stearic acid	C18:0	%	581	11.24	1.64	7.30	16.30	2.26	0.84 ± 0.18
Oleic acid	C18:1	%	585	52.32	2.87	44.40	59.70	5.14	0.61 ± 0.18
Linoleic acid	C18:2	%	584	2.35	0.48	1.20	3.80	0.03	0.13 ± 0.11
Linolenic acid	C18:3	%	585	0.13	0.04	0.10	0.20	0.0002	0.11 ± 0.10
Saturated fatty acids✝	SFA	%	586	39.78	3.05	32.60	48.20	4.98	0.53 ± 0.18
Unsaturated fatty acids‡	USFA	%	586	60.22	3.05	51.80	67.40	4.98	0.53 ± 0.18
Monounsaturated fatty acids§	MUFA	%	586	57.74	3.02	49.50	64.70	5.21	0.57 ± 0.18
Polyunsaturated fatty acids¶	PUFA	%	583	2.47	0.51	1.30	4.00	0.03	0.11 ± 0.10
Ratio between USFA and SFA	US/S		586	1.53	0.19	1.07	2.06	0.02	0.48 ± 0.17

✝ SFA = sum of C14:0, C16:0 and C18:0.

‡ USFA = sum of C14:1, C16:1, C18:1, C18:2 and C18:3.

§ MUFA = sum of C14:1, C16:1 and C18:1.

¶ PUFA = sum of C18:2 and C18:3.

**Table 2:** Descriptive statistics of water‐soluble compounds in meat and in moisture

Traits	Abbreviations	Trait values in meat (µmol/g meat)					Trait values in moisture (µmol/g moisture)				
		*n*	Mean	SD	Min	Max	n	Mean	SD	Min	Max
Free amino acids											
Aspartic acid	Asp	587	0.07	0.03	0.01	0.15	586	0.14	0.06	0.01	0.31
Glutamic acid	Glu	582	0.67	0.12	0.35	1.07	582	1.48	0.25	0.81	2.27
Asparagine	Asn	585	0.27	0.05	0.15	0.43	584	0.60	0.11	0.33	0.91
Serine	Ser	581	0.67	0.13	0.38	1.10	580	1.46	0.26	0.72	2.25
Glutamine	Gln	579	2.79	0.73	1.01	5.12	581	6.10	1.48	2.07	10.89
Histidine	His	587	0.35	0.11	0.16	0.64	584	0.77	0.24	0.35	1.49
Glycine	Gly	585	0.92	0.20	0.45	1.51	587	2.01	0.42	1.09	3.22
Threonine	Thr	581	0.45	0.08	0.25	0.71	579	0.99	0.17	0.56	1.52
β−alanine	β‐Ala	587	0.17	0.04	0.08	0.27	584	0.36	0.07	0.17	0.57
Arginine	Arg	582	0.48	0.09	0.28	0.74	582	1.06	0.18	0.56	1.60
Alanine	Ala	584	3.19	0.53	1.64	4.78	585	7.00	1.01	4.21	10.08
Taurine	Tau	582	1.11	0.35	0.25	2.20	584	2.44	0.79	0.52	4.87
Tyrosine	Tyr	584	0.38	0.08	0.22	0.60	582	0.83	0.15	0.44	1.30
Valine	Val	577	0.65	0.16	0.33	1.19	578	1.43	0.34	0.69	2.58
Methionine	Met	583	0.37	0.07	0.20	0.57	581	0.80	0.14	0.47	1.25
Tryptophan	Trp	586	0.13	0.03	0.05	0.22	586	0.29	0.07	0.12	0.46
Phenylalanine	Phe	584	0.42	0.08	0.25	0.69	583	0.93	0.17	0.50	1.48
Isoleucine	Ile	583	0.44	0.09	0.25	0.68	583	0.96	0.18	0.53	1.54
Leucine	Leu	583	0.88	0.17	0.46	1.39	581	1.92	0.34	0.98	2.98
Lysine	Lys	584	0.54	0.10	0.24	0.84	583	1.20	0.22	0.54	1.89
Proline	Pro	584	0.40	0.17	0.04	0.89	583	0.87	0.36	0.10	1.94
Total free amino acids	TFAA	583	15.40	2.25	9.38	22.57	583	33.72	4.25	23.01	45.93
Peptides											
Carnosine	Car	585	9.65	1.81	4.56	15.03	583	21.02	3.16	12.55	30.00
Anserine	Ans	585	2.09	0.46	1.02	3.49	584	4.57	0.91	2.23	7.26
Total peptides	TP	585	11.75	2.07	5.83	18.05	583	25.59	3.55	16.21	36.07
Nucleotides											
Inosine 5'‐monophosphate	IMP	584	0.39	0.21	0.01	1.03	584	0.85	0.42	0.04	2.09
Inosine	Ino	586	0.81	0.15	0.35	1.23	583	1.76	0.28	0.93	2.56
Hypoxanthine	Hx	581	2.04	0.30	1.07	3.00	581	4.46	0.63	2.59	6.39
Total nucleotides	TN	582	3.24	0.41	2.09	4.48	583	7.07	0.59	5.40	8.98
Sugars											
Ribose	Rib	584	1.18	0.22	0.60	1.80	585	2.58	0.41	1.53	3.81
Fructose	Fru	582	1.52	0.44	0.54	2.97	584	3.34	0.94	1.16	6.22
Mannose	Man	583	1.23	0.40	0.39	2.45	585	2.71	0.87	0.71	5.35
Glucose	Glc	581	5.48	1.01	2.79	8.71	584	12.08	2.14	7.06	18.61
Glycerol	Glyce	583	5.42	0.84	3.11	7.83	586	11.96	2.06	6.20	17.80
*myo*‐inositol	*myo*‐Ino	582	0.54	0.12	0.24	0.94	581	1.19	0.29	0.51	2.18
Total sugars	TS	583	15.41	2.43	8.74	22.98	585	33.88	5.31	18.90	49.46

## Results

### Heritabilities and genetic correlations of carcass traits, general composition and fatty acid composition

Descriptive statistics and heritabilities of carcass traits, general composition, and fatty acid composition are presented in Table 1. Heritability estimates for CW and BMS were calculated to be 0.28 and 0.22, respectively, whereas heritability estimates for MOIS, CF and CP were 0.24, 0.25 and 0.19, respectively, and 0.61 and 0.57 for oleic acid (C18:1) and monounsaturated fatty acids (MUFA), respectively. With the exception of linoleic acid (C18:2), linolenic acid (C18:3) and polyunsaturated fatty acids (PUFA) (for which heritability estimates were about 0.1 for each), the heritability estimates for fatty acid composition ranged from moderate to high (0.48–0.85). The genetic and phenotypic correlations among CW, BMS, MOIS, CF, CP, C18:1 and MUFA are presented in Table 3. The genetic and phenotypic correlations between each pair of traits displayed similar trends. The genetic and phenotypic correlations among general composition and those among fatty acid composition were very high (close to −1.0 or 1.0), and the genetic and phenotypic correlations of BMS with general composition were also relatively high (absolute values of 0.73 to 0.80 for genetic correlations and 0.68 to 0.69 for phenotypic correlations). On the other hand, the genetic and phenotypic correlations between CW and several other traits, BMS and fatty acid compositions, and general compositions and fatty acid compositions, were comparatively low (0.09 to 0.27 for genetic correlations and 0.07 to 0.26 for phenotypic correlations).

**Table 3:** Genetic and phenotypic correlations among carcass weight (CW), beef marbling standard (BMS), moisture (MOIS), crude fat (CF), crude protein (CP), oleic acid (C18:1) and monounsaturated fatty acids (MUFA)

Traits✝	CW	BMS	MOIS	CF	CP	C18:1	MUFA
CW		0.17	−0.17	0.14	−0.24	0.27	0.21
		(0.36)	(0.35)	(0.35)	(0.39)	(0.28)	(0.29)
BMS	0.20		−0.73	0.74	−0.80	0.12	0.09
	(0.05)		(0.19)	(0.18)	(0.17)	(0.33)	(0.33)
MOIS	−0.26	−0.68		−0.99	0.99	−0.20	−0.21
	(0.04)	(0.03)		(0.00)	(0.02)	(0.30)	(0.31)
CF	0.24	0.69	−0.99		−0.99	0.18	0.18
	(0.05)	(0.02)	(0.00)		(0.01)	(0.30)	(0.31)
CP	−0.17	−0.69	0.93	−0.95		−0.10	−0.15
	(0.04)	(0.02)	(0.01)	(0.00)		(0.34)	(0.34)
C18:1	0.15	0.09	−0.07	0.08	−0.10		0.93
	(0.05)	(0.05)	(0.05)	(0.05)	(0.05)		(0.03)
MUFA	0.17	0.10	−0.11	0.12	−0.14	0.95	
	(0.05)	(0.05)	(0.05)	(0.05)	(0.05)	(0.01)	

✝ Upper diagonal is genetic correlation, lower diagonal is phenotypic correlation. Standard errors are shown in parentheses.

### Effect of aging period, heritabilities and genetic correlations of water‐soluble compounds in meat and moisture

Water‐soluble compounds are generally expressed in the form of content of meat (Feidt *et al.* 1996; Cornet & Bousset 1999; Koutsidis *et al.* 2008a, 2008b), but given that taste is based on dissolved water‐soluble compounds reacting with the taste buds (Iida *et al.* 2015), we elected to investigate the genetic effects of the concentrations of water‐soluble compounds in both meat and moisture. Descriptive statistics of water‐soluble compounds are shown in Table 2.

Significance of aging effect and heritabilities of water‐soluble compounds in meat and moisture are presented in Table 4. Regarding aging effect, 15 of 36 traits in meat and 20 of 36 traits in moisture were significantly affected by aging period, with *P*‐values < 0.05. The heritability estimates for water‐soluble compounds in both meat and moisture displayed similar trends for each trait. For glutamine (Gln), alanine (Ala), Tau, Ans, IMP, inosine (Ino) and *myo*‐Inositol (*myo*‐Ino), heritability estimates were moderate (0.25–0.66 in meat and 0.31–0.59 in moisture), whereas those of the other water‐soluble compounds were below 0.30 in both meat and moisture. The heritability estimate for Tau was the highest (0.66 in meat and 0.59 in moisture). In our cattle population, most of the traits exhibiting a significant effect of aging had low heritability (< 0.30), with the exception of IMP, for which the heritability estimate was 0.47 in both meat and moisture.

**Table 4:** Significance of aging effect, heritability and genetic (r_G_) and phenotypic (r_P_) correlations for water‐soluble compounds in meat and in moisture

Traits✝	Trait values in meat (µmol/g meat)			Trait values in moisture (µmol/g moisture)			Correlations between water‐soluble compounds in meat and moisture§	
	*P*‐value‡	Genetic variance	Heritability	*P*‐value ‡	Genetic variance	Heritability		
							r_G_	r_P_
Free amino acids								
Asp	<0.001	0.0000000003	0.00 ± 0.00	<0.001	0.0000000001	0.00 ± 0.00	n.e.	n.e.
Glu	0.003	0.002	0.17 ± 0.11	<0.001	0.01	0.11 ± 0.09	0.73 ± 0.24	0.79 ± 0.02
Asn	0.003	0.00001	0.01 ± 0.06	<0.001	0.000000003	0.00 ± 0.00	n.e.	n.e.
Ser	0.014	0.002	0.12 ± 0.09	<0.001	0.01	0.16 ± 0.12	0.79 ± 0.18	0.83 ± 0.01
Gln	0.821	0.13	0.25 ± 0.14	0.350	0.63	0.31 ± 0.15	0.93 ± 0.06	0.92 ± 0.01
His	0.080	0.001	0.10 ± 0.08	0.437	0.003	0.08 ± 0.08	0.83 ± 0.17	0.92 ± 0.01
Gly	0.027	0.006	0.20 ± 0.11	0.226	0.013	0.10 ± 0.09	0.95 ± 0.28	0.83 ± 0.01
Thr	0.265	0.001	0.17 ± 0.10	0.234	0.002	0.08 ± 0.09	0.99 ± 0.42	0.80 ± 0.02
β‐Ala	0.006	0.0001	0.10 ± 0.09	0.007	0.001	0.22 ± 0.12	0.99 ± 0.13	0.85 ± 0.01
Arg	0.140	0.001	0.10 ± 0.09	0.012	0.004	0.13 ± 0.11	0.69 ± 0.30	0.80 ± 0.02
Ala	0.185	0.08	0.34 ± 0.14	0.074	0.37	0.39 ± 0.15	0.88 ± 0.09	0.77 ± 0.02
Tau	0.628	0.08	0.66 ± 0.17	0.419	0.36	0.59 ± 0.17	0.99 ± 0.01	0.95 ± 0.01
Tyr	0.005	0.001	0.20 ± 0.11	<0.001	0.004	0.19 ± 0.12	0.84 ± 0.13	0.83 ± 0.01
Val	0.123	0.003	0.18 ± 0.11	0.048	0.013	0.17 ± 0.12	0.95 ± 0.07	0.87 ± 0.01
Met	0.009	0.001	0.22 ± 0.11	0.001	0.004	0.25 ± 0.13	0.83 ± 0.12	0.83 ± 0.01
Trp	<0.001	0.00002	0.03 ± 0.06	<0.001	0.0001	0.03 ± 0.06	n.e.	n.e.
Phe	0.004	0.001	0.21 ± 0.11	<0.001	0.005	0.20 ± 0.12	0.84 ± 0.13	0.84 ± 0.01
Ile	0.001	0.001	0.12 ± 0.09	<0.001	0.004	0.14 ± 0.10	0.75 ± 0.21	0.83 ± 0.01
Leu	0.103	0.004	0.15 ± 0.10	0.006	0.006	0.05 ± 0.08	0.70 ± 0.30	0.83 ± 0.01
Lys	<0.001	0.001	0.11 ± 0.09	<0.001	0.004	0.09 ± 0.10	0.69 ± 0.33	0.83 ± 0.01
Pro	0.343	0.001	0.06 ± 0.07	0.384	0.008	0.07 ± 0.08	0.98 ± 0.05	0.96 ± 0.00
TFAA	0.077	0.86	0.19 ± 0.11	0.002	1.93	0.12 ± 0.10	0.71 ± 0.27	0.70 ± 0.02
Peptides								
Car	0.260	0.45	0.17 ± 0.10	0.560	1.67	0.22 ± 0.12	0.77 ± 0.17	0.82 ± 0.02
Ans	0.173	0.08	0.40 ± 0.16	0.094	0.29	0.36 ± 0.16	0.94 ± 0.04	0.89 ± 0.01
TP	0.310	0.53	0.15 ± 0.10	0.576	1.69	0.18 ± 0.11	0.71 ± 0.23	0.79 ± 0.02
Nucleotides								
IMP	0.004	0.02	0.47 ± 0.15	0.004	0.07	0.47 ± 0.15	0.99 ± 0.01	0.98 ± 0.00
Ino	0.131	0.01	0.33 ± 0.14	0.103	0.04	0.45 ± 0.16	0.91 ± 0.06	0.86 ± 0.01
Hx	<0.001	0.02	0.23 ± 0.12	<0.001	0.08	0.23 ± 0.12	0.90 ± 0.10	0.75 ± 0.02
TN	0.571	0.03	0.20 ± 0.11	0.071	0.07	0.22 ± 0.12	0.62 ± 0.26	0.62 ± 0.03
Sugars								
Rib	0.231	0.01	0.16 ± 0.11	0.028	0.02	0.14 ± 0.09	0.80 ± 0.16	0.83 ± 0.01
Fru	0.888	0.03	0.14 ± 0.11	0.949	0.03	0.04 ± 0.07	0.92 ± 0.12	0.94 ± 0.01
Man	0.510	0.02	0.18 ± 0.13	0.223	0.05	0.09 ± 0.10	0.91 ± 0.10	0.94 ± 0.01
Glc	0.737	0.18	0.22 ± 0.13	0.230	0.22	0.06 ± 0.08	0.70 ± 0.32	0.82 ± 0.02
Glyce	0.007	0.13	0.23 ± 0.12	<0.001	0.43	0.13 ± 0.10	0.75 ± 0.21	0.76 ± 0.02
myo‐Ino	0.879	0.005	0.37 ± 0.14	0.890	0.03	0.39 ± 0.14	0.97 ± 0.03	0.90 ± 0.01
TS	0.331	0.98	0.20 ± 0.12	0.013	1.60	0.08 ± 0.08	0.72 ± 0.28	0.74 ± 0.02

✝ Abbreviations of water‐soluble compounds are shown in Table 2.

‡ *P*‐value of significance by an incremental Wald F statistics analysis for aging effect.

§ n.e., not estimable.

The combinations of water‐soluble compounds in meat and moisture were analyzed using the multi‐trait animal model. The genetic and phenotypic correlations between the traits in meat and moisture are presented in Table 4. Correlations for Asp, asparagine (Asn) and tryptophan (Trp) were not estimable due to very low degrees of genetic variance. For the other traits, all water‐soluble compounds in meat had highly positive genetic correlations with those in moisture (0.62–0.99). The phenotypic correlations of water‐soluble compounds in meat with those in moisture were also highly positive (0.62–0.98) for all traits.

### Genetic and phenotypic correlations of water‐soluble compounds with CW, BMS and MUFA

The genetic and phenotypic correlations of water‐soluble compounds with CW, BMS and MUFA in meat are shown in Table 5. Asp, Asn and Trp were excluded because of their low genetic variances. The phenotypic correlations of water‐soluble compounds with CW and MUFA were very low (absolute values of 0.01 to 0.34 for CW and absolute values of 0.01 to 0.20 for MUFA). On the other hand, the genetic correlations of water‐soluble compounds with CW and MUFA were low to high (absolute values of 0.02 to 0.84 for CW and 0.06 to 0.98 for MUFA). The phenotypic correlations of water‐soluble compounds with BMS were low to moderate (absolute values of 0.04 to 0.60), whereas the genetic correlations of water‐soluble compounds with BMS were low to high (absolute values of 0.05 to 0.99). Most of the water‐soluble compounds were positively correlated with MUFA at the genetic level, but negatively with CW and BMS. Water‐soluble compounds with moderate heritability, such as Gln, Ala, Tau, Ans, IMP and *myo*‐Ino, were low to moderately correlated with CW (absolute values of 0.02 to 0.65), BMS (absolute values of 0.08 to 0.43) and MUFA (absolute values of 0.06 to 0.36), with the exception of the genetic correlation of Tau with CW, which was negative (−0.84).

**Table 5:** Genetic (r_G_) and phenotypic (r_P_) correlations of water‐soluble compounds in meat with carcass weight (CW), beef marbling standard (BMS) and monounsaturated fatty acids (MUFA)

Traits✝	CW		BMS		MUFA	
	r_G_	r_P_	r_G_	r_P_	r_G_	r_P_
Free amino acids						
Glu	−0.42 ± 0.39	−0.05 ± 0.05	−0.53 ± 0.36	−0.35 ± 0.04	0.79 ± 0.27	0.01 ± 0.05
Ser	−0.52 ± 0.41	−0.27 ± 0.04	−0.63 ± 0.44	−0.33 ± 0.04	0.53 ± 0.40	−0.06 ± 0.05
Gln	0.21 ± 0.36	−0.04 ± 0.05	−0.08 ± 0.42	−0.28 ± 0.04	−0.28 ± 0.30	0.01 ± 0.05
His	−0.66 ± 0.36	−0.04 ± 0.05	−0.78 ± 0.46	−0.08 ± 0.05	−0.36 ± 0.37	0.04 ± 0.05
Gly	−0.64 ± 0.30	−0.21 ± 0.04	−0.41 ± 0.38	−0.24 ± 0.04	−0.06 ± 0.33	−0.01 ± 0.05
Thr	−0.52 ± 0.36	−0.22 ± 0.04	−0.74 ± 0.42	−0.24 ± 0.04	0.46 ± 0.34	0.01 ± 0.05
β‐Ala	0.21 ± 0.47	−0.18 ± 0.04	−0.11 ± 0.52	−0.22 ± 0.04	0.10 ± 0.44	−0.05 ± 0.05
Arg	−0.10 ± 0.46	−0.23 ± 0.04	0.05 ± 0.51	−0.27 ± 0.04	0.98 ± 0.21	−0.04 ± 0.05
Ala	−0.02 ± 0.32	−0.13 ± 0.05	−0.16 ± 0.34	−0.32 ± 0.04	0.06 ± 0.29	0.07 ± 0.05
Tau	−0.84 ± 0.21	−0.05 ± 0.05	−0.39 ± 0.28	−0.13 ± 0.05	−0.13 ± 0.24	0.10 ± 0.06
Tyr	−0.48 ± 0.34	−0.20 ± 0.04	−0.79 ± 0.35	−0.35 ± 0.04	0.36 ± 0.34	−0.10 ± 0.05
Val	−0.31 ± 0.37	−0.17 ± 0.04	−0.53 ± 0.40	−0.28 ± 0.04	0.70 ± 0.29	−0.03 ± 0.05
Met	−0.48 ± 0.34	−0.20 ± 0.04	−0.89 ± 0.33	−0.36 ± 0.04	0.39 ± 0.33	−0.07 ± 0.05
Phe	−0.54 ± 0.34	−0.15 ± 0.04	−0.99 ± 0.43	−0.33 ± 0.04	0.42 ± 0.33	−0.04 ± 0.05
Ile	−0.44 ± 0.43	−0.14 ± 0.04	−0.91 ± 0.49	−0.32 ± 0.04	0.52 ± 0.37	−0.04 ± 0.05
Leu	−0.30 ± 0.40	−0.14 ± 0.04	−0.80 ± 0.35	−0.34 ± 0.04	0.55 ± 0.34	−0.04 ± 0.05
Lys	−0.24 ± 0.46	−0.12 ± 0.04	−0.79 ± 0.45	−0.26 ± 0.04	0.78 ± 0.32	0.11 ± 0.05
Pro	−0.59 ± 0.52	0.01 ± 0.04	−0.27 ± 0.59	−0.09 ± 0.04	0.22 ± 0.54	0.01 ± 0.05
TFAA	−0.64 ± 0.36	−0.18 ± 0.04	−0.54 ± 0.33	−0.40 ± 0.04	0.12 ± 0.36	0.01 ± 0.05
Peptides						
Car	−0.44 ± 0.36	−0.23 ± 0.04	−0.59 ± 0.31	−0.44 ± 0.04	0.08 ± 0.37	−0.11 ± 0.05
Ans	−0.02 ± 0.33	−0.07 ± 0.05	−0.43 ± 0.31	−0.35 ± 0.04	0.36 ± 0.26	0.20 ± 0.05
TP	−0.39 ± 0.38	−0.22 ± 0.04	−0.67 ± 0.29	−0.47 ± 0.04	0.22 ± 0.39	−0.05 ± 0.05
Nucleotides						
IMP	−0.27 ± 0.28	−0.24 ± 0.05	−0.19 ± 0.30	−0.25 ± 0.05	−0.18 ± 0.25	−0.15 ± 0.05
Ino	−0.09 ± 0.32	−0.18 ± 0.05	−0.46 ± 0.29	−0.44 ± 0.04	0.10 ± 0.30	−0.03 ± 0.05
Hx	0.22 ± 0.34	0.07 ± 0.05	−0.19 ± 0.39	−0.37 ± 0.04	0.55 ± 0.30	0.07 ± 0.05
TN	−0.36 ± 0.38	−0.16 ± 0.04	−0.72 ± 0.21	−0.60 ± 0.03	0.18 ± 0.35	−0.03 ± 0.05
Sugars						
Rib	0.44 ± 0.37	0.08 ± 0.05	−0.50 ± 0.32	−0.44 ± 0.04	0.44 ± 0.42	0.04 ± 0.05
Fru	−0.30 ± 0.41	0.21 ± 0.04	−0.49 ± 0.37	−0.27 ± 0.04	0.53 ± 0.42	0.02 ± 0.05
Man	−0.08 ± 0.39	0.34 ± 0.04	−0.49 ± 0.36	−0.24 ± 0.04	0.81 ± 0.47	0.10 ± 0.05
Glc	−0.23 ± 0.38	0.19 ± 0.05	−0.52 ± 0.33	−0.36 ± 0.04	0.73 ± 0.37	0.06 ± 0.05
Glyce	−0.05 ± 0.37	0.11 ± 0.05	−0.63 ± 0.28	−0.24 ± 0.04	0.26 ± 0.33	0.14 ± 0.05
myo‐Ino	0.65 ± 0.27	0.10 ± 0.05	0.37 ± 0.34	0.04 ± 0.05	−0.07 ± 0.28	−0.06 ± 0.05
TS	−0.08 ± 0.38	0.23 ± 0.04	−0.61 ± 0.28	−0.38 ± 0.04	0.50 ± 0.37	0.09 ± 0.05

✝ Abbreviations of water soluble compounds are shown in Table 2.

## Discussion

### Genetic effects of carcass traits, general composition and fatty acid composition

Several researchers have reported low to moderate heritability estimates in Japanese Black cattle for CW (0.37–0.75) and BMS (0.49–0.61) (Shojo *et al.* 2006; Inoue *et al.* 2008; Osawa *et al.* 2008; Nogi *et al.* 2011). In regards to fatty acid composition, researchers have reported moderate to high heritability estimates for C18:1 (0.54–0.78) and MUFA (0.66–0.68), and low genetic correlations between carcass traits and fatty acid composition in Japanese Black cattle (Inoue *et al.* 2008, 2011; Nogi *et al.* 2011). To our knowledge, there have been no reports of heritability estimates and genetic correlations between the general composition factors of meat determined by chemical analysis, and thus the present study is the first to assess the genetic effects of MOIS, CF and CP.

In the present study, the heritability estimates of CW and BMS were lower than those of previous studies, whereas the heritability estimates for fatty acid composition were consistent with those of previous reports. One of the reasons for our lower estimates of heritability of CW and BMS may be the limited grades of the samples used in the present study, as 567 out of the 587 total samples were graded between A3 and A5. Another possible reason is that about one‐half of the samples used in this study are mainly derived from two grandsires. For measuring chemical traits, the samples were obtained at one specific meat processing plant and included only a limited number of sampling years (i.e. 2011 to 2013). Thus, the size of our population was small. Japanese Black cattle are characterized by high levels of marbling, because of improvements in marbling scores through generations of artificial selection. Therefore, the limited number of grandsires could be responsible for the low genetic variance of the selection traits, such as CW and BMS. On the other hand, the unselected traits, such as fatty acid composition, may retain high genetic variance, even with a restricted number of grandsires. Our results also indicated that it is possible to capture the genetic variance for unselected traits, such as water‐soluble compounds, even for small populations and low numbers of grandsires. However, a larger sample size and similar evaluations of other cattle breeds are needed to obtain more reliable estimates.

### Physiological and environmental factors of water‐soluble compounds

Water‐soluble compounds, such as free amino acids, peptides and sugars, are affected by factors such as diet (Koutsidis *et al.* 2008a), type of muscle (Cornet & Bousset 1999), post mortem conditioning (Koutsidis *et al.* 2008b), as well as feeding system factors, such as fattening period (Okumura *et al.* 2007) and concentration of serum vitamin A (Oka *et al.* 1998). Water‐soluble compounds with moderate heritability estimates for physiological and environmental factors were as follows.

Free amino acids: the heritability estimates for Gln, Ala and Tau were moderate to high, with Tau yielding the highest heritability value of the water‐soluble compounds. Tau is synthesized from methionine or cysteine (HuxTable 1992) and is largely unaffected by proteolysis (Cornet & Bousset 1999); in fact, Moya *et al.* (2001) reported that Tau concentrations in pork did not change during aging. Furthermore, Iwamoto *et al.* (2009) and Iwamoto *et al.* (2010) reported that Tau concentrations in the meat of Japanese Black steers were not influenced by the duration of the fattening period nor the dietary protein level. Our results showed that there is no significant effect of aging on Tau concentrations, suggesting that Tau concentrations could be affected by genetic rather than environmental effects. It has been shown that concentrations of Gln and Ala are not influenced by either the duration of the fattening period or the dietary protein level, but may be affected during aging (Koutsidis *et al.* 2008b; Iwamoto *et al.* 2010), but we did not detect any significant effect of aging on either Gln or Ala.

Peptides: the concentration of Car yielded low heritability estimates, but that of Ans yielded moderate heritability estimates. Car and Ans are dipeptides containing β‐alanine (β‐Ala) and histidine (His), which yielded low heritability estimates in the present study. Although Car and Ans are progressively hydrolyzed to β‐Ala and His in post mortem conditioning (Lawrie & Ledward 2006), their concentrations remain stable over the first 7 days of conditioning (Moya *et al.* 2001; Koutsidis *et al.* 2008b). However, it has been reported that Car concentration decreases after 14 days of conditioning (Koutsidis *et al.* 2008b) and is influenced by levels of dietary protein (Iwamoto *et al.* 2010), and thus the heritability estimate for Car should be less than that for Ans. The heritability estimates reported in the present study and those reported by Mateescu *et al.* (2012) are consistent with this logic: the heritability estimates for Car and Ans reported by Mateescu *et al.* (2012) were 0.38 and 0.53, respectively, and were 0.17 and 0.40, respectively, in our study.

Nucleotides: the heritability estimates recorded for IMP, Ino and hypoxanthine (Hx) were 0.47, 0.33 and 0.23, respectively, in meat; and 0.47, 0.45 and 0.23, respectively, in moisture. Ino is a derivative of IMP, and in turn then degrades to Hx (Koutsidis *et al.* 2008b). IMP is an upper cascade product of nucleotide metabolism that yielded higher heritability estimates than those of its degradation products in the present study. Koutsidis *et al.* (2008a) reported that IMP concentration is not influenced by diet; however, other studies have shown that IMP content is affected by the length of the fattening period (Iwamoto *et al.* 2009), the aging period (Iida *et al.* 2008a,) and by post mortem conditioning (Koutsidis *et al.* 2008a, 2008b). In addition, Koutsidis *et al.* (2008a) demonstrated that Ino concentrations were affected both by diet and by genetic effects, specifically the interaction between breed and diet. In the present study, there were significant associations between aging period and both IMP and Hx; the trend of the best linear unbiased estimator (BLUE) for aging period revealed that IMP concentrations in meat decreased, whereas concentrations of Hx in meat increased in response to longer aging period duration (Fig. S1).

Sugars: the concentration of *myo*‐Ino yielded moderate heritability estimates. This compound is a sugar alcohol and an important component of phospholipids (Kuksis 2003). The concentration of *myo*‐Ino can be altered during the aging process, and can be affected by both the length of the fattening period and the level of dietary protein (Koutsidis *et al.* 2008b; Iwamoto *et al.* 2010). However, Koutsidis *et al.* (2008a) also demonstrated that *myo*‐Ino is influenced by genetic effects, as evidenced by the interaction between breed and diet. In our study, there was no significant effect of aging on *myo*‐Ino concentrations.

In the present study, we found the aging period to be one of the most important factors affecting water‐soluble compounds. Most of the traits for which there was a significant effect of aging had low heritability, and some traits with moderate heritability were not affected by aging period, with the exception of IMP. Iida *et al.* (2016) demonstrated that IMP concentrations declined significantly between days 10 to 20. In this study, the aging period was limited to the period of 16 to 19 days, and the effect of aging period was then neglected as a fixed effect in the statistical analyses. As a result, genetic variance could exist and be captured using samples collected within a specific interval of the aging period, even the aging period affect for IMP.

### Genetic effects of water‐soluble compounds

Little information is available regarding the genetic effects of water‐soluble compounds in the meat of beef cattle; to our knowledge, only Mateescu *et al.* (2012) has attempted to do so, by evaluating the genetic parameters for peptides in the Longissimus muscle of Angus cattle, for which they reported low to moderate heritability estimates for concentrations of carnitine (0.015), creatine (0.434), creatinine (0.070), Car (0.383) and Ans (0.531). In comparison then, the present study reports the genetic effects of a relatively comprehensive range of water‐soluble compounds. Most of the traits we analyzed yielded relatively low heritability estimates, and they tend to be affected by the aforementioned environmental factors; however, heritability estimates were moderate for some traits, such as Gln, Ala, Tau, Ans, IMP, Ino and *myo*‐Ino. In addition, phenotypic correlations of water‐soluble compounds with CW, BMS and MUFA were generally low, but genetic correlations were low to high.

In regard to meat quality, the palatability of beef is primarily evaluated by the sensory characteristics such as taste, tenderness, juiciness and aroma. However, sensory characteristics are largely subjective and difficult to measure, and require the use of trained taste panels to assess many complex parameters involved in the eating experience. As such, obtaining a large number of samples for these traits is difficult. To our knowledge, in regard to investigating genetic effects based on sensory evaluations, only a few studies have reported low heritabilities, including those of Gill *et al.* (2010) (ranging from 0.06 to 0.16) and Mateescu *et al.* (2015) (ranging from 0.06 to 0.25). Therefore, it is difficult to improve meat quality by relying on sensory characteristics alone. Another approach is to incorporate sensory‐related traits such as chemical traits as indicators. In that case, it would be necessary to gain a better understanding of the relationship between sensory characteristics and chemical traits, and to evaluate the genetic effects of chemical traits. Our study contributed to the evaluation of genetic effects on chemical traits, and demonstrated that some of these traits have moderate heritability. As such, meat quality could be improved by using those traits with moderate heritability as indicators, if these traits are also associated with sensory characteristics. Thus, in addition to evaluating genetic effects, the relationships between sensory characteristics and chemical traits must be investigated further.

## Conclusion

In the present study, genetic parameters for 54 carcass and chemical traits, such as general composition, fatty acid composition and water‐soluble compounds (including free amino acids, peptides, nucleotides and sugars) in Japanese Black cattle were assessed. Most of the water‐soluble compounds yielded low heritability estimates; however, some traits have moderate heritability estimates and high genetic correlation with CW, BMS and MUFA. These findings form a basis for further studies on the genetic effects of chemical traits in beef.

## Supporting information


**Figure S1** Changes in the best linear unbiased estimator (BLUE) of inosine 5′‐monophosphate (IMP) and hypoxanthine (Hx) in meat during the aging period from 16 to 19 days.

Supporting info item
