# Supplementary material for: Estimates of genetic parameters for chemical traits of meat quality in Japanese black cattle
Source: Anim Sci J. 2016 May 5;88(2):203–12. doi: 10.1111/asj.12622 (PMC5298003; doi:10.1111/asj.12622)
Supplement: Supplementary file 1 — Supporting info item [file ASJ-88-203-s001.pdf]

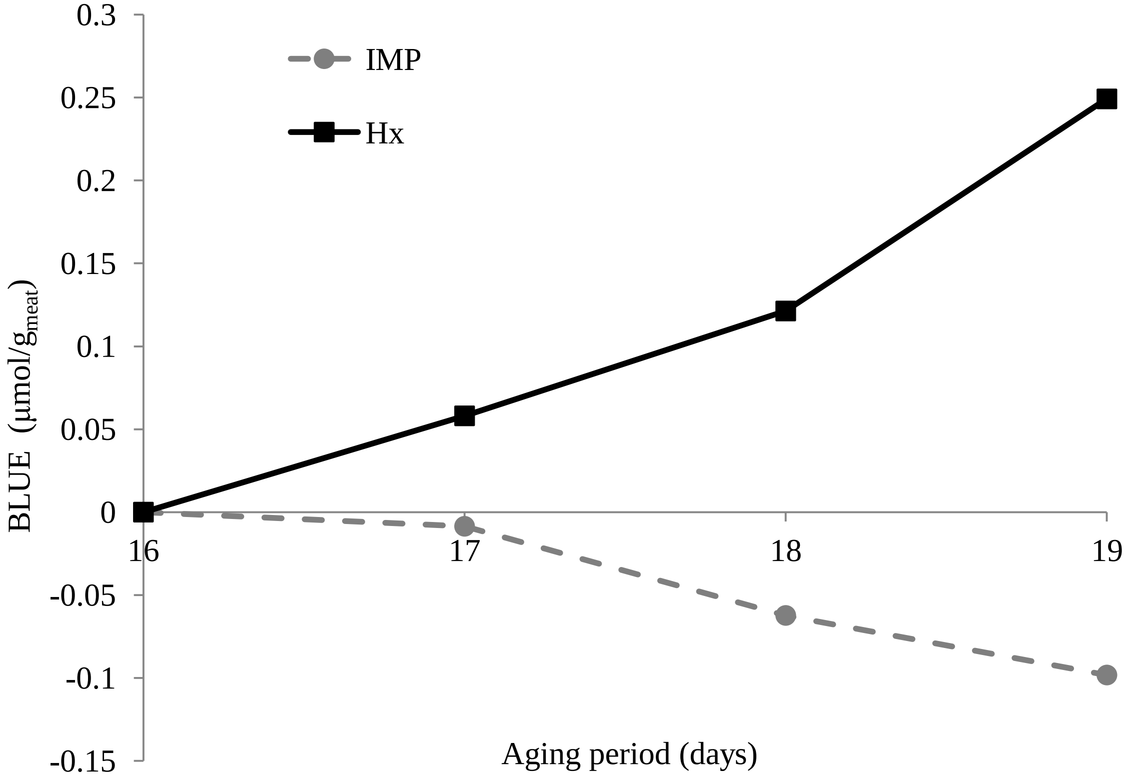

Figure S1. Changes in the best linear unbiased estimator (BLUE) of inosine 5′-monophosphate (IMP) and hypoxanthine (Hx) in meat during the aging period from 16 to 19 days.

FigureS1
